# Non-coding RNAs in congenital heart disease and placental development: Bridging molecular mechanisms to clinical biomarkers and therapies

**DOI:** 10.1016/j.ncrna.2025.08.006

**Published:** 2025-09-01

**Authors:** Haoxuan Wang, Xinzhe Chen, Yinghui Li, Shudan Xiao, Tianqi Teng, Sumin Yang, Kun Wang, Meihua Zhang

**Affiliations:** a Key Laboratory of Birth Regulation and Control Technology of National Health Commission of China, Shandong Provincial Maternal and Child Health Care Hospital Affiliated to Qingdao University, Jinan, 250014, China; b Department of Cardiovascular Surgery, Institute of Chronic Diseases, The Affiliated Hospital of Qingdao University, Qingdao, 266003, Shandong, China

**Keywords:** ncRNA, CHDs, Placenta, Epigenetics, Heart-placenta axis, Biomarker

## Abstract

Non-coding RNAs (ncRNAs) have emerged as pivotal regulators of gene expression, orchestrating embryonic development and disease pathogenesis. This review synthesizes current knowledge on the origin, biogenesis, and functional diversity of ncRNAs, with a focus on their regulatory crosstalk in congenital heart disease (CHD) and placental development. The fetal heart-placenta axis, a bidirectional signaling network essential for cardiogenesis and placental morphogenesis, is spatiotemporally modulated by ncRNAs through epigenetic and post-transcriptional mechanisms. Through precise regulation of cardiac cell differentiation, angiogenesis, and trophoblast invasion, ncRNAs maintain developmental homeostasis, whereas their dysregulation disrupts these processes, contributing to CHD pathogenesis and positioning them as promising biomarkers. Collectively, this review establishes ncRNAs as molecular bridges between the fetal heart-placenta axis and clinical translation, underscoring their dual utility as diagnostic biomarkers for CHD and modifiable targets to correct placental maldevelopment, thereby advancing precision therapies for congenital disorders.

## 1 Introduction

Congenital heart disease (CHD), characterized by congenital malformations of heart valves, walls or blood vessels, is the most common type of birth defect, affecting ∼1 % of live births and accounting for over 19 % of deaths from non-communicable diseases [1,2]. Tetralogy of fallot (TOF), ventricular septal defect (VSD), atrial septal defect (ASD), hypoplastic left heart syndrome and other phenotypes are among the various types of CHD [[3], [4], [5]]. However, in most cases, the underlying cause of these disorders is still unknown despite decades of study that has mostly focused on genetic etiology [6]. Some studies have recently demonstrated that unexplained CHD may be the primary cause of undiscovered mechanisms involving noncoding genetic and epigenetic factors [7]. In addition, the involvement of placental abnormalities in CHD has garnered significant attention [8].

The placenta, developed from the trophectoderm, is the first crucial organ responsible for material exchange, metabolism, barrier function and other physiological processes linking the fetus and the mother. The healthy development of the embryonic heart and other organs depends on appropriate early placental development. As observed in both animal models and clinical studies, abnormal development of placenta and heart frequently coexist [9,10]. And the placenta-heart axis describes a dynamic interdependence between fetal cardiac function and placental development, mediated through umbilical vasculature [11]. Accounts of studies in vivo have suggested that normal placentation is a prerequisite for healthy cardiovascular development [[12], [13], [14]]. However, little is known about the exact cellular and molecular processes underlying the placenta's role in promoting embryonic development, particularly cardiovascular morphogenesis.

RNA that does not encode a protein but is important for post-transcriptional control of gene expression is referred to as non-coding RNA (ncRNA). ncRNAs are classified into various types based on their length, structure, and location. These classifications include PIWI-interacting RNA (piRNA), circular RNA (circRNA), long non-coding RNA (lncRNA), and microRNA (miRNA). In recent years, a growing body of research has confirmed the significant roles of ncRNAs in cardiovascular diseases and placental abnormalities [15,16]. These findings not only underscore the complexity of ncRNAs as post-transcriptional regulators but also provide novel insights into the mechanisms underlying the placenta-heart axis. This review aims to comprehensively review and prospectively explore the latest advancements in ncRNA research related to CHD and placental abnormalities, with the goal of driving further breakthroughs in this thriving field.

## 2 Definition and biogenesis of ncRNAs

### 2.1 Biogenesis and origin of miRNA

MicroRNAs are a subclass of small and evolutionarily preserved regulatory ncRNAs that mature to approximately 20–26 nucleotides (nt) in length. Numerous miRNAs have been identified in humans, animals, and plants since they were initially identified in the worm *Caenorhabditis elegans* [17]. The evolutionary conservation of miRNA structure and biogenesis pathways is critical for understanding their gene regulatory mechanisms.

The miRNA-encoding genes are scattered over the genome, with a large fraction arranged in clusters that encompass several miRNAs. The expression of miRNAs is linked to the transcription and processing of host genes since they are partially encoded inside and may overlap with protein-coding or noncoding genes. Nevertheless, separate transcriptional units can potentially be the source of miRNAs [18,19]. Transcription, nuclear maturation, export, and cytoplasmic processing are all steps in the complex, multistage process that miRNAs go through during biogenesis before becoming functional RNAs that fulfill their assigned functions.

**Fig. 1:**
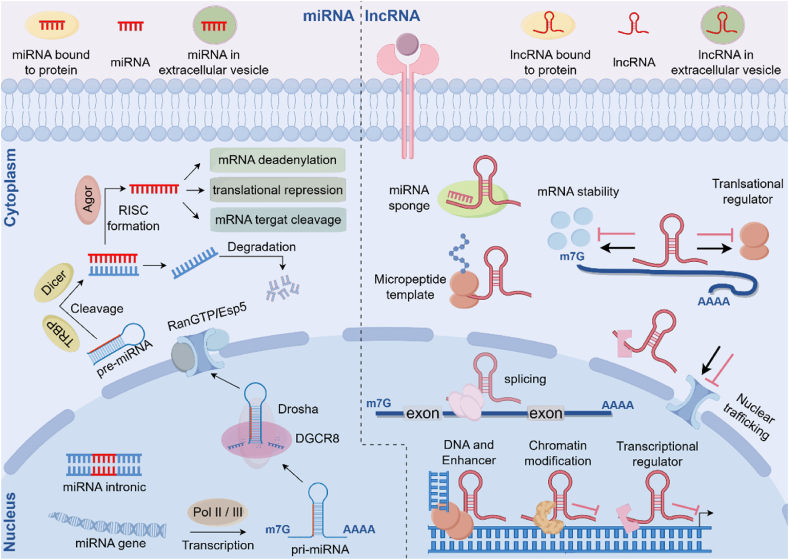
**Biogenesis and function of miRNAs and lncRNA.** MiRNAs undergo endonucleolytic processing after being transcribed from lengthier precursors or from introns. They are integrated into the RISC to control the expression of target transcripts by translational repression or degradation (left). LncRNAs serve as regulators of mRNA translation and degradation, protein templates, or sponges for other transcripts or proteins in the cytoplasm. Through their interactions with transcriptional regulators, chromatin-modifying complexes, and DNA, they regulate gene expression in the nucleus (right). Both miRNAs and lncRNAs are secreted and detected in the extracellular space, where they can attach to proteinous binding partners or be stabilized in vesicles.

RNA polymerase (Pol II) transcribes a significant fraction of miRNA genes as polycistronic transcripts, while RNA Pol III is responsible for a smaller fraction [20,21]. This transcription produces large primary miRNA transcripts (pri-miRNAs) that share characteristics with mRNAs, including 5′ capping and 3′ polyadenylation. Pri-miRNAs adopt a hairpin structure which contains the mature miRNA sequence within its stem region [22,23]. Pri-miRNAs undergo two crucial processing steps. The initial nuclear cleavage is mediated by the Microprocessor complex, which includes the DGCR8 protein and the RNase III enzyme Drosha. DGCR8 recognizes the pri-miRNA and recruits Drosha to cleave it [24], generating a hairpin-shaped precursor miRNA (pre-miRNA) of approximately 60–70 nucleotides (nt). This pre-miRNA is then exported to the cytoplasm by Exportin-5 in complex with Ran-GTP [25,26]. In the subsequent processing step, the RNase III endonuclease Dicer, in complex with TAR RNA-binding protein (TRBP), cleaves the pre-miRNA close to the terminal loop, releasing short double-stranded RNA duplexes. The miRNA-induced silencing complex (miRISC) is assembled when these duplexes quickly attach to Ago proteins [23,27] (Fig. 1).

The mature miRNA strand, referred to as the guide strand, remains within the miRISC, whereas the complementary strand of the duplex, often termed the passenger strand, undergoes degradation. The choice of which strand serves as the guide strand is a dynamic process that exhibits both spatial and temporal variations. Numerous variables influence this decision, such as the intrinsic properties of the precursor miRNA and the involvement of other processing factors or complexes [28,29]. Moreover, miRNAs are generated from tiny hairpin introns known as mirtrons through a distinct mechanism termed the noncanonical pathway. In this case, splicing bypasses the Drosha-mediated step, directly generating the intron lariat containing the pre-miRNA sequence. This intron lariat is then debranched, releasing a linear RNA that folds into the characteristic hairpin structure of a pre-miRNA [30]. The canonical biogenesis route is subsequently used to further process these pre-miRNAs. To further increase the variety of their sources, pre-miRNAs can also be derived from small nucleolar RNAs (snoRNAs) or transfer RNA (tRNA) precursors [31,32]. In conclusion, the biogenesis of miRNAs is a complex and intricate process involving multiple crucial steps and regulatory factors. As a crucial mechanism of post-transcriptional gene regulation, miRNA biogenesis ensures the accurate synthesis of functional miRNAs.

### 2.2 LncRNA

#### 2.2.1 Characteristics and biogenesis of lncRNA

Despite being one of the least explored ncRNA classes, lncRNAs have garnered increasing attention, with novel classes, attributes, and roles continually being uncovered and annotated each year. Commonly, lncRNAs refer to transcripts exceeding 200 nt in length and devoid of protein-coding signatures. This length criterion serves as a practical biophysical delimiter, distinguishing lncRNAs from other types of RNA. For instance, infrastructural RNA species, such as the primate-specific small RNA [33], Y RNAs [34], vault RNAs [35], as well as non-canonical small RNAs generated by post-transcriptional processing and promoter-associated RNAs [36], all fall outside of this length criterion and are thus distinguished from lncRNAs. Notably, the definition of lncRNAs is not absolute, as exemplified by the remarkable lengths attained by some, such as Airn and KCNQ1OT1 transcripts, which surpass 90 kb [21]. Meanwhile, other ncRNAs, like 7SK [37,38] (approximately 330 nt) and 7SL [39] (approximately 300 nt), may exceed this length criterion yet are not classified as lncRNAs. Depending on 7SL, the signal recognition particle directs proteins to cell membranes, whereas 7SK regulates transcription pausing and termination, especially at enhancers. Additionally, 7SL shares evolutionary relationships with the tiny interspersed nuclear elements of the rodent B1 (approximately 135 nt) and the widespread monkey Alu (approximately 280 nt) [[40], [41], [42]].

Compared to the mRNA sequences that encode the proteome, the majority of lncRNAs are less conserved across species. However, this reduced conservation does not necessarily imply functional irrelevance. In fact, lncRNAs display a more nuanced pattern of conservation, often surpassing that of introns or intergenic regions [43,44], suggesting that they play crucial roles in cellular processes. Initial studies perceived the majority of the mammalian genome, including lncRNA loci, as evolving neutrally based on the divergence rates of ancient repeats between human and mouse genomes. Yet, emerging evidence has challenged this notion, revealing that transposable elements are commonly appropriated as functional components in gene expression and structure, forming promoters, regulatory networks, exons, and splicing sites for protein-coding genes and lncRNAs [[45], [46], [47]]. Consequently, these elements cannot be relied upon as reliable indicators of neutral evolution in lncRNAs.The function of lncRNA is dynamic, as highlighted by the rapid evolution of its regulatory sequences, including promoters, which occurs due to relaxed structural and functional constraints compared to protein-coding sequences [48,49]. Cell lineage specificity is a common characteristic of many lncRNAs, with variations in their complement and sequences potentially contributing significantly to species diversity. The existence of multiple exons, promoters, distinctive chromatin signatures, alternative splicing, and control over morphogens and transcription factors are some characteristics of functional genes that lncRNAs display despite their poor sequence conservation [43,[50], [51], [52]]. They also display comparable levels of promoter conservation to protein-coding genes and retain orthologous functions despite rapid sequence evolution [50,53].

Similar to the creation of protein-coding transcripts, the biogenesis of lncRNAs mostly takes place in the nucleus, especially in the chromatin-associated fraction [50,54]. Histone modifications frequently epigenetically mark lncRNA promoters, and transcription factors control them to either enhance or inhibit gene expression [55]. Similar to mRNAs, RNA Pol II transcribes many lncRNAs, whereas Pol III preferentially transcribes certain lncRNA promoter topologies [50,56]. Another characteristic that unites lncRNAs and their protein-coding counterparts is post-transcriptional processing. A sizable fraction of lncRNAs undergo 3′ polyadenylation and 5′ capping [57]. Almost all lncRNAs contain standard splice sites, which result in the production of at least two isoforms of transcripts, each primarily made up of two exons. Unlike mRNAs, which are usually confined to specific cellular locations, lncRNAs can be located in diverse cellular compartments. A certain proportion of lncRNAs are exported to the cytoplasm during biogenesis and processing, whereas the majority of non-coding transcripts stay in the nucleus and are directed to chromatin [50]. The last phase of lncRNA biogenesis is the formation of thermodynamically stable structures, which is an essential feature of these molecules. The functional activity of lncRNAs at the basic structural level depends on Watson-Crick base pairing, which enables direct interaction with other RNA molecules such as mRNAs and miRNAs or with DNA [58]. The discovery that lncRNAs exhibit more structured conformations compared to their protein-coding counterparts [59] suggests that their structural adaptability may play a pivotal role in enabling the formation of binding sites for interacting with other DNA or RNA molecules and proteins [60]. This emphasizes how closely the structure of lncRNAs and their biological roles interact. The function of lncRNAs and RNA alterations in functional plasticity, as well as the process of lncRNA folding, which includes determining auxiliary components and clarifying the structure-function links within non-coding transcripts, are still being studied. Therefore, this complexity a significant hurdle for future studies that seek to identify new lncRNA regulation mechanisms.

### 2.3 Characteristics and biosynthesis of circRNA

CircRNAs are present across diverse organisms. Specifically, they are highly abundant in eukaryotes. These molecules are evolutionarily conserved and can be distinctive to particular cell types or specific developmental stages [61,62]. CircRNAs exhibit a unique structure where the 3′ and 5′ ends are covalently linked, forming a continuous single-stranded loop, in contrast to the linear structure of linear RNA. Due to their circular structure, circRNAs have decreased susceptibility to exonuclease activity and are inappropriate for further processing, leading to a high degree of stability, in contrast to linear RNAs [63]. The size of circRNAs spans from 100 nt up to more than 4 kilobases (kb), encompassing single or multiple exons, or intronic sequences [21]. Because circRNAs are stable, their expression levels may be higher than those of their corresponding linear mRNAs, and they often show no association with the expression levelsof their host genes [64,65]. With respect to their subcellular distribution, circRNAs are found primarily in the cytoplasm, but they can also be found in the nucleus and, like lncRNAs, they can bind to DNA and form circR-loops [66,67].

**Fig. 2:**
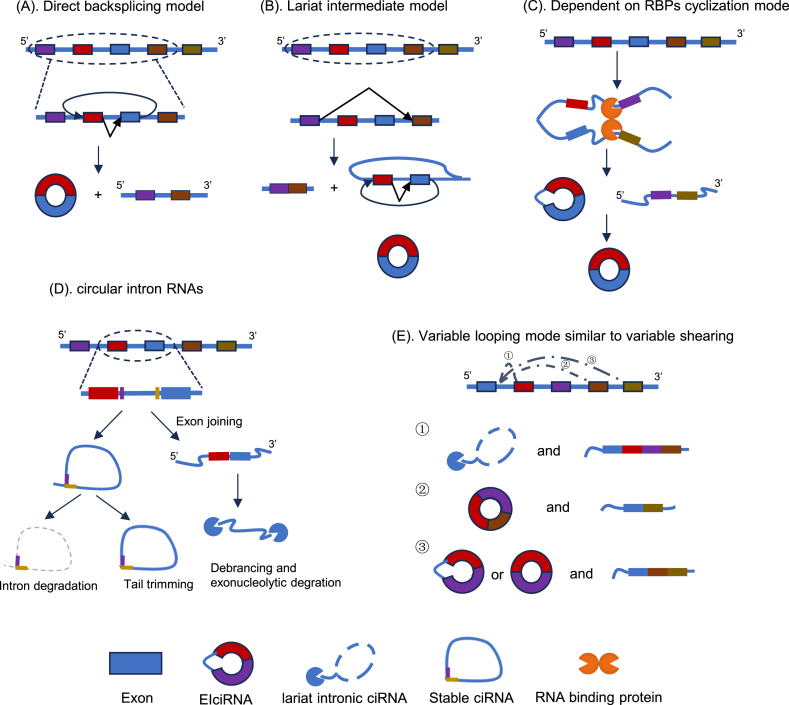
**The splicing model in the formation of circular RNAs.** (A) Linear RNA is produced from an exon–(multiple) intron-exon intermediate (box) after direct backsplicing first produces a circRNA. (B) Canonical The first step is splicing, which results in a linear RNA without introns. Meanwhile, back-splicing is performed on a long intron lariat that includes the skipped exons. (C) RNA binding proteins (RBPs) recognize motifs in the flanking introns at both ends of the cyclized exon. RBPs promote the closeness and linkage of the flanking introns to form a loop by binding to their particular motifs to form a dimer. (D) Between the splicing donor and the branch point, the removed intron lariat can avoid degradation and create a ciRNA inside itself. (E) Three fundamental types of alternative splicing in circRNAs that contain numerous exons.

CircRNA, originating from exons, introns, or a combination of both [68], exhibit a diverse and complex circularization mechanism (Fig. 2). The production of circRNA primarily relies on specific splicing events, with exonic circRNA generation being particularly crucial. They can form circular products through a process known as back splicing, which involves the reverse connection of the upstream 3′ splice site with the downstream 5′ splice site [61,69]. This process may be facilitated by inverted repeats repeats typically found in the introns adjacent to the exons [70]. Alternatively, exonic circRNAs can also be produced through a mechanism that involves exon skipping. During this process, a lariat intermediate, which contains both the exons and the introns, is formed as part of the splicing pathway. Subsequently, this intermediate undergoes further processing to remove the intron and circularize the exon-containing portion. [71,72]. This precursor undergoes self-splicing, leading to circularization of the lariat structure upon intron removal [73]. Furthermore, alternative back-splicing generates single-exon circRNAs [74]. The nucleus contains intronic circRNAs, which are made up of one or more introns and are produced from linear RNA. The biosynthesis of intronic circRNAs relies crucially on specific elements: an 11-nucleotide C-rich sequence near the branch point and a 7-nucleotide GU-rich motif near the 5′ splice site [62]. Another type, nuclear exon-intron circRNAs, incorporate both intronic and exonic sequences. The presence of flanking repeat sequences can enhance their circularization [75]. Notably, circRNAs not only exhibit diverse circularization patterns but also function as miRNA/protein sponges or interact with mRNAs/RBPs [76]. Emerging evidence reveals that RNA-binding motif protein 24 mediates miRNA sequestration by circRNAs. [77]. In the next section, a detailed exploration of the functions and mechanisms of circRNAs will be presented.

## 3 Function and related mechanisms of miRNA, LncRNA, and circRNA

### 3.1 miRNA function and related mechanisms

The defining characteristic of miRNA function lies in their capacity to silence gene expression through precise sequence-specific binding to their target RNAs. Within the seed region of miRNA, Watson-Crick base pairing largely facilitates this recognition process, which aligns with complementary nucleotides situated within the 3′ untranslated region (3′ UTR) of the target RNA [21,23]. Additionally, a minor fraction of miRNAs exhibits the ability to regulate gene expression by recognizing and targeting distinct segments of other mRNAs [78].

The degree of complementarity determines the binding strength and suppression mechanism. Moreover, this intricate regulatory process is also influenced by other elements like the quantity, location, and flanking regions, secondary structures and accessibility of target sites, as well as RNA-induced sequence modifications. Apart from the interaction between naked RNA molecules, miRNA and RNA targeting are partially determined by connection with the effector proteins in miRISC [22,23]. The RISC complex is activated by miRNAs upon inclusion, focusing its particular activity on the targeted location. Therefore, the intricate relationship between the miRNA and RISC results in the suppression of the target, either by disrupting the translational machinery or by encouraging the nucleolytic destruction of the mRNA. Ago proteins, which compete with elements that promote ribosomal subunit recruitment, translation start, or the breakdown of the translational apparatus, are responsible for this translational suppression. In order to attract endonucleolytic and exonucleolytic enzymes to carry out the process [79], target degradation proceeds either by means of deadenylation occurring at the 3′ end or decapping taking place at the 5′ end.

miRNAs exhibit other functions in addition to their typical post-transcriptional activity. For instance, miRNAs recruit the appropriate ribonucleoprotein complexes to cause target translation. Furthermore, a number of studies have discovered that the physiological function of miRNAs is somewhat dependent on their localization in the nucleus [80,81]. Moreover, some miRNAs and adjacent Ago proteins have the ability to gather in the nucleus, so they can affect the biogenesis of additional miRNAs and RNA species [21]. Nevertheless, nothing is now known about the underlying mechanisms.

Outside of the cell, miRNAs can also be found in the extracellular environment, including plasma, urine, breast milk, blood and other body fluids, where they are referred to as circulating miRNAs [82]. Because they bind to proteinaceous carriers or are encapsulated in a variety of vesicle and microparticle forms, circulating miRNAs are persistent and impervious to RNase degradation. In this regard, miRNAs serve as adaptable signaling molecules that transmit genetic information between tissues and cells [83]. Circulating miRNAs have enormous promise as vital diagnostic biomarkers because of the close relationship between miRNA expression profiles and disease states, especially cardiovascular disorders [84].

### 3.2 LncRNA function

LncRNAs exhibit remarkable versatility in their genomic origins, capable of being transcribed from diverse locations across the genome in both sense and antisense orientations. These transcripts can arise from introns and exons within protein-coding genes, often overlapping with coding sequences, as well as from intergenic regions. Additionally, pseudogenes, inactive copies of functional genes, can serve as templates for lncRNA transcription [85]. Illustrating their functional significance, the lncRNA AFAP1-AS1, transcribed in antisense orientation, exemplifies this diversity and has been shown to promote myocardial infarction progression by modulating the miR-512-3p/RTN3 axis [86]. Another source of lncRNAs that emphasizes their conservation across species is transcribed ultraconserved regions, or T-UCRs. Additionally, lncRNAs are transcribed from telomeres, which contain repetitive sequences at the ends of chromosomes, and from centromeric repeats, producing centromeric lncRNAs. Ribosomal DNA loci, encompassing the promoter and pre-rRNA antisense regions, also contribute to the lncRNA repertoire. Moreover, lncRNAs are associated with promoters, leading to the formation of promoter-associated lncRNAs, and with enhancers, generating enhancer RNAs. Finally, they can be found within 3′UTRs of genes, giving rise to UTR-associated RNAs [87].

Through direct interactions with DNA, lncRNA form R-loops, which occur throughout the genome but are especially abundant at the locations of active genes. These R-loops are essential for controlling gene expression and chromatin structure, but they can seriously jeopardize genomic integrity, especially at the crucial stage of DNA replication [88]. As well as associating with DNA, lncRNAs are also of great significance in other aspects. For example, the human HOX loci contain sequences that encode for more than 200 lncRNAs [89]. Among them stands out HOTAIR, a trans-regulatory lncRNA also known as HOX Antisense Intergenic RNA [90]. The HOXC locus is the source of HOTAIR, which functions as a repressor of the HOXD locus despite the latter being on a different chromosome. Notably, PRC2 and LSD1, two chromatin-modifying complexes, are physically interacting with HOTAIR [91].

Unlike chromatin-modifying proteins, which must re-enter the nucleus to function, lncRNAs can act directly at their site of transcription. This unique property renders them ideally suited for cis-regulation, allowing them to fine-tune gene expression in a more precise and efficient manner [21]. The 108-kb ncRNA Airn is exclusively transcribed in antisense orientation from the paternal allele of the Igf2r cluster. This transcript exhibits characteristic features of ncRNAs, including substantial length, low evolutionary conservation, inefficient splicing, and short half-life [92]. The interaction between Airn and the paternal Igf2r cluster provides another example of antisense/sense pairing [[92], [93], [94]]. In order to mute Slc22a2, Igf2r, and Slc22a3 on the paternal allele, Airn functions in cis. Histone methyltransferase EHMT2 is brought to the chromosomal locus to accomplish this silencing, which results in allelic silencing [94] and H3K9 methylation. Notably, a stable RNA product is not necessary for the silence of Airn during the repression of Igf2r. Rather, the recruitment of Pol II to its target site is disrupted by the simple transcription of Airn. Moreover, the Igf2r promoter is covered by the Airn sequence. Airn inhibits the start of transcription on the Igf2r promoter [93] by being continually transcribed. Recent studies demonstrate that lncRNA TMEM9B-AS1 regulates ribosomal biogenesis by stabilizing the mRNA of the transcription factor MYC. This occurs through its direct binding to the RNA-binding protein IGF2BP1 [95].

Numerous lncRNAs primarily exert their functions within the nucleus by regulating gene expression. MALAT1, for example, is a very prevalent lncRNA that is mostly found in the nucleus, especially around the edges of nuclear speckles. MALAT1 indirectly regulates AKT3 via competitive binding with miR-150-5p [96]. Anril facilitates the recruitment of PRC2 and PRC1, chromobox 7 within the polycomb repressive complex 1 binds to ANRIL, and NEAT1 contributes to the formation of paraspeckles [97,98]. As well as functioning in the nucleus, lncRNA can interact with other RNAs in the cytoplasm. CDK19 expression is indirectly up-regulated by hotair sponging miR-222-3p expression [99]. Cell survival is improved by PANDA, the 5′-capped and polyadenylated nonspliced lncRNA that transcribes antisense to the CDKI-coding gene CDKN1A, by preventing the transcription factor NF-YA from occupying target gene promoters. The expression of both PANDA and CDKN1A is p53-dependent, however it's interesting to note that PANDA enhances cell survival by imitating the apoptotic genes expression program. In contrast, CDKN1A mediates cell cycle arrest by encoding a cell cycle inhibitor. To stabilize and maintain its abundance, TINCR directly interacts with a particular set of differentiation-associated mRNAs through the TINCR box, a 25-nt motif that is enriched in both the target mRNAs and TINCR itself. Concurrently, TINCR and STAU1, a known RNA-binding protein, form a complex that permits interaction with mRNAs and is essential for their stabilization [100]. Notably, recent studies reveal novel lncRNA-driven mechanisms with therapeutic implications. A prime example is ZFAS1, which was recently identified to activate DICER1 transcription and stabilize its mRNA—effectively mimicking DICER1 function to modulate microRNA networks [101].

### 3.3 CircRNA function

CircRNAs exhibit a diverse array of functions, including acting as miRNA sponges, interacting with mRNAs, serving as templates for translation, and engaging in protein interactions. For instance, circRNAs, widely recognized as a novel type of competing endogenous RNA (ceRNA) that decoys relevant miRNAs, serve as key regulators of miRNA target genes. by influencing their bio-functions. With a superior ability to bind miRNAs compared to other ceRNA, circRNA is also termed a “super sponge”. An excellent illustration of this is the circular RNA sponge for miR-7 (ciRS-7), which effectively absorbs miR-7 through more than 70 binding sites [61,71]. Human ciRS-7 mimics the phenotype resulting from miR-7 knockdown, demonstrating its sponge-like function [61]. Resistant to miRNA-mediated instability, ciRS-7 serves as an effective inhibitor of miR-7 activity, leading to an increase in the transcript levels of miR-7 targets. Additionally, high levels of endogenous interaction between ciRS-7 and miR-7 have been observed in mouse brains, particularly in hippocampal and neocortical neurons [71]. Another RNA circle, originating from the sex-determining region Y, comprises 16 binding sites for miR-138 and functions as a sponge for this miRNA, thereby upregulating the expression of its target genes [102]. Recent research has highlighted the critical function of nuclear circular intronic RNAs (ciRNAs) in human cell transcriptional regulation, specifically their capacity to cis-act to control the transcription of their parent genes. Nuclear ciRNAs dramatically increase Pol II-mediated transcription, as evidenced by the interaction of ci-mcm5, ci-sirt7, and ci-ankrd52 with RNA Pol II and the marked decrease in gene transcription that results from their absence [62]. Additionally, circCANX forms a ternary complex with P53 mRNA and RNA helicase upstream frameshift 1 that mediates P53 mRNA degradation via the nonsense-mediated mRNA decay pathway [103].

Additionally, a growing body of data indicates that exon-intron circular RNAs (EIciRNAs), including EIciPAIP2 and EIciEIF3J, interact at the promoter region with RNA Pol II and the U1 small nuclear ribonucleoprotein (snRNP) to stimulate the transcription of their parent genes. The preserved introns of each of these EIciRNAs have a binding site for U1 small nuclear RNA (snRNA). The amount of EIciRNA-U1 snRNP complexes anchored at the promoter of the coding gene decreases when the RNA-RNA connection is disrupted because it inhibits the binding between Pol II and EIciRNA.Ultimately, this reduction in complex formation results in decreased gene transcription levels [75]. Additionally, because it contains binding sites for one or more proteins, CircRNA can act as a protein decoy to control gene expression. Studies have demonstrated connections between circRNA and RNA Pol II as well as Ago proteins [62,104]. CircMBL further supports the decoy function of circRNA by sequestering the MBL protein and preventing it from binding to other targets [105]. Due to their inherent stability, circRNAs can serve as “scaffolds” for RNA-binding proteins, enabling stable interactions and the binding of multiple proteins. For example, circFoxo3 forms a ternary complex (circFoxo3-p21-CDK2) with both p21 and CDK2, which stops cell cycle progression and suppresses the biological action of CDK2 [106]. Compelling evidence has been found to support the idea that circRNAs can encode proteins. CircRNAs' involvement in translation processes is confirmed by the fact that they can be translated efficiently both in vitro and in vivo when they have an internal ribosome entry site [107,108]. Moreover, the translation of circRNA in human cells can occur via rolling circle amplification, independently of traditional translational elements [109]. A survey unveiled that a substantial number of circRNAs utilize a novel cap-independent translation mechanism [110]. Meanwhile, Legnini and Pamudurti et al. also presented in vitro and in vivo evidence supporting the existence of cap-independent translation in circRNAs [111,112]. Emerging studies highlight the critical role of circRNAs in regulating alternative splicing, with recent work revealing their functional interplay with splicing factors in cancer pathways. For example, chemotherapy-induced splicing factors QKI and CELF4 directly govern the biogenesis of circFLNB through alternative splicing. Mechanistically, this circRNA suppresses colorectal cancer tumorigenesis in vivo and in vitro by sponging miR-3127-3p to activate the MOB1B/Hippo signaling axis [113]. Furthermore, circRNAs possess the capability to serve as enhancers of proteins. They accomplish this by attracting proteins to particular loci or subcellular compartments, forming complexes with proteins [106], or serving as scaffolding for proteins [114]. Their co-localization is facilitated by this recruitment, and this in turn affects interactions between proteins [115]. Mitochondria-encoded circRNAs (mecciRNAs), a newly identified subclass, critically regulate mitochondrial homeostasis by targeting TNF receptor-associated protein 1 and cyclophilin D [116]. This interaction modulates mitochondrial permeability transition pore gating and mitochondrial reactive oxygen species release [117].

In summary, we have offered an exhaustive and thorough review of ncRNA, encompassing its definition, classification, structure, function, and other pertinent aspects in the preceding chapters. This work is foundational and indispensable for subsequent chapters delving into the role of ncRNA in CHD and placental development.

## 4 Development of embryonic heart and placenta

The etiology of CHD is commonly attributed to abnormal development of the embryonic heart, which can be induced by placental dysfunction. Consequently, in this section, the crucial phases of the placental and cardiac development of the embryo have been reviewed.

### 4.1 Embryonic heart development

The embryonic heart development process of vertebrates is continuous and can be artificially divided into different stages. For instance, the anterior lateral plate mesoderm establishes paired heart-forming fields during the process of identifying myo- and endocardial precursor cells. The paired heart-forming fields then merge in front of the growing foregut to produce the linear heart tube. The cardiac looping follows, and then the creation of the chambers, the septation, and the heart valves [118]. In particular, the identification of cardiac progenitor cells inside the anterior lateral plate mesoderm can be used to identify the first stage of human embryonic heart development. This process occurs from the zygote stage and continues until roughly the fifteenth day of human embryonic development, a timeframe that corresponds to embryonic day 7.5 in mice. These progenitor cells condense during this time to create two lateral heart primordia, which are made up of endocardial and myocardial progenitors. The two primordia then unite at their front borders to form what is called the “cardiac crescent” [119,120]. During this crucial developmental stage, a number of important transcription factors, including NKX2-5, MEF2, GATA4 family members, and SRF, are essential for the proliferation and differentiation of cardiomyocytes [119,121,122]. Heart and neural crest derivatives-expressed 1 (Hand1) and Hand2 are the two genes that subsequently become active in the developing heart. The entire linear heart tube exhibits Hand2 expression, whereas Hand1 expression is restricted to the regions of the first cardiac field that will eventually form the left ventricle. Hand2 is thus more strongly expressed in the derivatives of the second cardiac field that will give rise to the right ventricle. Furthermore, the early development of the heart depends on a number of genes. Take for example in the cardiac cell lineage, mesoderm posterior 1 (Mesp1) is one of the first genes produced that is essential for heart formation [123].

After the cardiac crescent forms, the bilateral heart primordia migrate medially by day 20 of human embryonic development (E8.5 in mice). They then join at the midline to form a heart tube composed of an external myocardial layer and an internal endocardial layer. At this stage, MESP1/2, GATA4/5/6, TBX5, and miles-apart are essential regulators for normal heart development [[120], [121], [122]]. Furthermore, during this period, the final differentiation of cardiomyocytes is regulated by signals from bone morphogenetic protein and fibroblast growth factor [120]. The cardiac tube in humans has looped rightward by day 30 of embryonic development (E10-12 in mice). The growth of the right ventricle and outflow tract is aided by cells from the second heart field throughout this looping phase. Important cardiac looping regulators include NKX2-5, SNAI1, PITX2, Hand1/2, XIRP1, and LEFTY1/2 [119,124]. Furthermore, during this crucial stage [119], the establishment of the left-right axis and left-right asymmetry depends on signals from NODAL and Hedgehog. Furthermore, it should be noted that LEFTY1 and LEFTY2 are antagonists of NODAL signaling [120]. Furthermore, it is important to remember that Wnt signaling is necessary for myocardial specification, cardiac morphogenesis, and the development of cardiac valves, among other aspects of cardiac development [118,119,124].

**Fig. 3:**
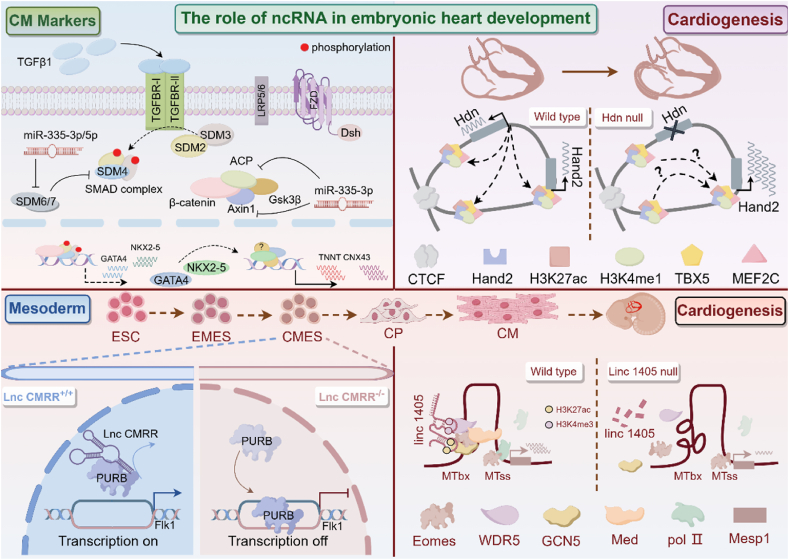
**The regulatory mechanism of ncRNA mediated early cardiac development.** The expression of cardiac differentiation markers is regulated by miR-335-3p and miR-335-5p (top left) [125]. The cis-located Hand2 gene is regulated by transcription of Handsdown to control the development of the embryonic heart (top right) [126]. By controlling the Purb/Flk1 axis, LncCMRR contributes significantly to cardiac differentiation (bottom left) [127]. To further coordinate Mesp1 expression and cardiac specification, lincRNA 1405 mediates an Eomes/WDR5/GCN5 regulatory complex (bottom right) [128].

It is evident that embryonic heart development is intricately regulated by a series of sophisticated mechanisms, encompassing specific transcription factors, growth factors, signaling pathways, and certain structural proteins. These regulatory elements play crucial roles at various stages of embryonic heart development, collectively ensuring the normal and orderly development of the heart into a fully functional organ (Fig. 3).

### 4.2 Placenta development

The critical initial stages of placental development encompass trophoblast lineage morphogenesis in the blastocyst, trophectoderm, blastocyst apposition and adhesion, implantation period and initial formation of the syncytiotrophoblast, villous formation and the villous trophoblast population, extravillous trophoblast subpopulations and trophoblast invasion, and also endoarterial trophoblast, endovenous and endolymphatic trophoblast [129].

In general, human placental development requires the trophoblast lineages of the placenta and the maternal endometrium to interact simultaneously. About five to six days after fertilization (DPF), the blastocyst's polar trophectoderm adheres to the receptive endometrial epithelium, starting the human placentation process. Embryonic positioning is associated with the receptor tyrosine kinase-like orphan receptor signaling pathway regulated by Wnt5 [130]. Trophectoderm cells then unite with neighboring cells to form a main syncytium. The endometrium changes into the specialized tissue known as the decidua119,120 when masses of syncytiotrophoblast start to infiltrate the gaps between endometrial cells at 6 to 8 DPF [131,132]. During the expansion of the primary syncytiotrophoblast, the blastocyst is enclosed by the lumen's closure, forming an implantation chamber. This process entails fluid pinocytosis by epithelial cells, potentially dependent on the ENaC sodium channel activation, downstream of SGK-1 tyrosine kinase signaling [133,134]. The remaining unfused trophectoderm cells that make up the blastocyst wall are now known as cytotrophoblast cells. Through fusion, these progenitor cells enlarge the chorionic plate's primary syncytiotrophoblast [135]. The whole gestational sac has been covered by the primary syncytiotrophoblast by the end of implantation. Fluid-filled lacunae form within the syncytial mass, and the syncytiotrophoblast develops into a complex network of trabeculae as they unite and grow. The decidual glands, which produce secretions that feed the syncytiotrophoblast, are eroded by these trabeculae [131,135].

Around 12 DPF, a crucial stage in placental development unfolds, marked by the proliferation and invasion of trophoblast cells. Primary villi are created when cytotrophoblast cells multiply and pierce the trabeculae. The cytotrophoblast shell, a new layer positioned between the villi and the decidua, is finally formed by these cells migrating laterally to form cell columns that anchor the placenta to the decidua [131]. Meanwhile, extravillous trophoblasts (EVT) begin their invasion of maternal spiral arteries, decidual veins, uterine glands, and lymphatic vessels, working to remodel these structures in support of placental function [136]. Both processes are integral to establishing a functional placental structure, with EVT focusing on maternal blood vessel remodeling and cytotrophoblast cells forming the anchoring and supportive layers [135].

### 4.3 Heart-placenta axis

Placenta-heart axis describes a dynamic interdependence between fetal cardiac function and placental development, mediated through umbilical vasculature. The two organs evolve synchronously, sharing key developmental mechanisms; thus, CHD frequently demonstrates co-occurrence with placental dysfunction. Consequently, CHD has been found to frequently coexist with placental dysfunction. We have outlined the crucial stages of embryonic heart and placental development, and damage to these developmental processes undoubtedly represents a potential factor contributing to CHD. Some studies showed that placental abnormality, such as reduction placental weight and villous vasculature, and immaturity placental villi, occurred at higher rates in CHD placentas [12,13]. Researchers have also demonstrated that morphogenesis of the heart and vascular system and placental dysfunction are linked in animal model [137]. Specifically, placental trophoblast-specific knockouts are a common cause of lethal CHD [138]. A common issue in placentally produced CHD models is abnormalities in the maternal blood-facing syncytiotrophoblasts in mice, which emphasizes the placenta and its early malfunction as a potentially important source of developing heart malformations [139]. Defects in the syncytiotrophoblast layer facing maternal blood are a major cause of developmental heart disease in placentally produced CHD models, according to a recent large-scale in vivo phenotyping study [140]. This research underscores the placenta's pivotal role in the etiology of CHD, highlighting the essential need to include placental factors in the investigation of CHD causes. Furthermore, Lee et al. provided further insights into the maternal-fetal interface organization and placental impact on embryonic development, focusing on syncytiotrophoblast and its derivative PIBF1. During the first trimester of pregnancy, syncytiotrophoblasts in the human placenta exhibit elevated expression of PIBF1, a progesterone immunomodulatory binding factor. Their findings revealed the regulatory role of PIBF1 in trophoblast syncytialization and its promotion of cardiovascular development [14].

## 5 The function of ncRNA in CHD

We have comprehensively summarized the roles and underlying mechanisms of miRNAs, lncRNAs, and circRNAs in the context of CHD over the recent years (Table 1).

**Table 1:** The function of ncRNA in CHDs.

Conditions	ncRNA name	Cell and animal model	Target	Function	References
CHD	miR-140/miR-195	Both	Mfn1/Mfn2	Reduce mitochondrial lengths and leading to mitochondrial fragmentation and CHD	[141]
CHD	miR-871-3p	Both	Megf8	Inhibit formaldehyde-induced inflammation and CHD	[142]
CHD	miR-222/miR-503	Both	FGF9/IGF2	Suppress the transcription of compact myocardial related growth factors	[143]
CHD	miR-153-3p	Both	βII Spectrin	Regulate cardiomyocyte apoptosis and inhibit formaldehyde -induced CHD	[144]
CHD	miR-375	Both	NOTCH	Disrupt cardiac development of Zebrafish	[145]
CHD	miR-29b-3p	Both	NOTCH2	Inhibit cardiomyocyte proliferation and induce cardiac malformation in zebrafish embryos	[146]
CHD	miR-144	In vitro	TBX1	Regulates cardiomyocyte apoptosis and cell cycle	[147]
CHD	miR-29c-3p	In vitro	Akt3	Inhibit the proliferation, and promote apoptosis and differentiation of P19 cells.	[148]
CHD	miR-592	Both	KCTD10	Negatively regulate the proliferation and apoptosis of embryonic endocardial cells	[149]
CHD	lncRNA NONMMUT063967.2	Both	FGFR2	Regulate the effects of ASXL3 gene mutations on mouse cardiomyocytes proliferation and apoptosis	[150]
CHD	pCharme	Both	MATR3	sustains developmental gene expression in fetal cardiomyocytes	[151]
CHD	lnc-TSSK2-8	In vitro	HSPA6 and CRYAB	Influence cardiac outflow tract development	[152]
CHD	FOXD3-AS1	In vitro	miR-150-5p	Negatively regulate cardiomyocyte apoptosis and protected cardiomyocytes from hypoxia-induced injury	[153]
CHD	Upperhand	Both	Hand2	Regulate mice right ventricle hypoplasia and embryonic lethality	[154]
CHD	CircRNA 105039	In vitro	miR-17	promotes cardiomyocyte differentiation	[155]
CHD	circ-RCCD	Both	MyD88	promoted cardiomyocyte differentiation	[156]
CCHD	lncRNA SNHG14	In vitro	miR-25-3p	protected cardiomyocytes against hypoxia-induced injury	[157]
ASD	miR-20b-5p	In vitro	TET2	Suppressed human embryonic stem cells-derived cardiac differentiation	[158]
ASD	Moshe	Both	Nkx2.5	Negatively regulate the expression of secondary heart field lineage genes.	[159]
TOF	miR-222	Both	DICER1/AGO2	TOF-related cardiac defects	[160]
TOF	lncRNA TBX5‐AS1:2	Both	TBX5	May be involved in TOF by affecting cell proliferation	[161]
TOF/CHD	FGD5-AS1	In vitro	miR-421	Negatively regulate the apoptosis of fetal heart cells and CHD-associated genes expression.	[162]
VSD	lncRNA MEG3	Both	miR-7-5p	inhibit cardiomyocytes autophagy	[163]

### 5.1 MicroRNA in CHD

During fetal development, structural or functional abnormalities of the heart or major vessels are referred to as CHD [164]. These anomalies are intricately linked to the fetal developmental environment and the precise regulation of various developmental stages. In this section, we will focus on the role of miRNA in CHDs, encompassing a thorough review and synthesis of their influence on cardiomyocytes, CHD animal model, fetal heart development, and lastly, their potential as diagnostic biomarkers.

Several miRNAs have been proven as regulatory molecules in cardiomyocytes. For example, miRNA-144 inhibited cardiomyocyte proliferation and triggered apoptosis by targeting TBX1 which is one of the core genes of CHD [147]. Overexpression of miRNA-29c-3p, upregulation in the serums of pregnant women carrying fetuses with CHD, suppressed cardiomyocytes proliferation and triggered apoptosis by inhibiting Akt3 [165]. Recent studies have shown that miRNA-20b-5p is upregulated in plasma samples from individuals with ASD, and further demonstrating that miRNA-20b-5p targets TET2, a positive regulation molecular in embryonic development, and 5-hydroxymethylcytosine to inhibit cardiac differentiation [158].

**Fig. 4:**
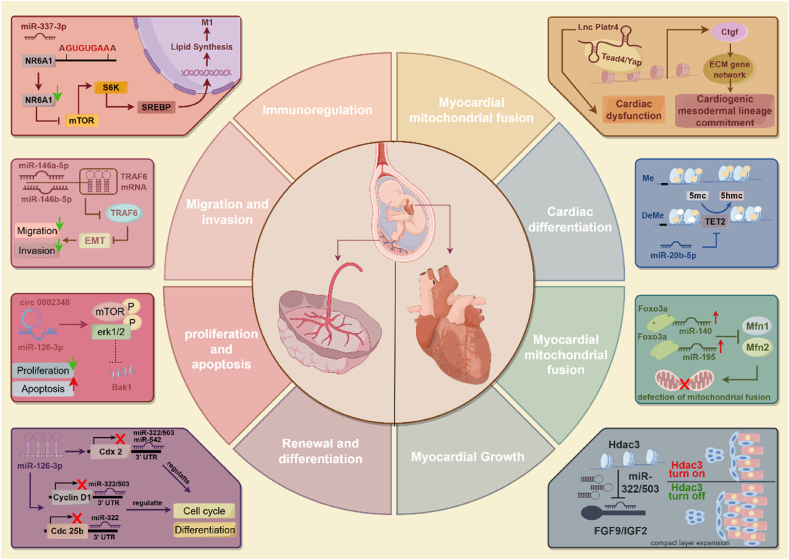
**Mechanisms of ncRNAs in different CHDs and placenta development.** Current studies have demonstrated that ncRNAs regulate a number of cell biology-related processes during the development of the heart and placenta, including immunoregulation, cell cycle, migration, invasion, apoptosis, and proliferation.

Other researchers have studied the effect of miRNA on CHD in vivo. Shen et al. found that miRNA-29c, microinjecting into zebrafish embryos, overexpression attenuated heart development in a dose-dependent manner, further experiments revealed that aforementioned function of miRNA-29c are performed through targeting Wnt4 [166]. By targeting NOTCH2, miRNA-29b-3p, an upregulated miRNA in the right ventricular outflow tract of CHD patients, caused cardiac deformity and dysfunction in zebrafish and suppressed cardiomyocyte proliferation [146]. Researchers have demonstrated that appropriate early embryonic heart development requires a progressive reduction in the expression of miR-222, which was found in TOF, one of the most prevalent complex CHD datasets [160]. They also evaluated the detrimental effects of elevated miR-222 expression in the developing heart. Meanwhile, Jiang et al. revealed that knockdown miR-222 and miR-503 enhances compact cardiac development by targeting FGF9 and IGF2 [143]. The majority of current research has shown that miR-140 and miR-195 shorten mitochondrial lengths, which causes fragmentation. Disruption of mitochondrial function also contributes to the development of CHDs [141]. Research at the cellular level and animal model has suggested a potential regulatory role of miRNAs in CHDS, underscoring their involvement in the intricate mechanisms underlying these conditions. Clinical evidence showed that abnormal expression of several ncRNAs are detected by generation sequence in CHD-related disease [167]. In serum samples from individuals with cyanotic CHD, miRNA-182 expression was downregulated; in vitro, miRNA-182 has a protective effect in cyanotic CHD models [168]. What's more, researchers have showed that miRNA-871-3p knockdown can inhibit formaldehyde-induced CHD in vivo and vitro [142]. These findings provide the evidence for interventions targeting ncRNA-mediated congenital heart defect and placental abnormality (Fig. 4).

### 5.2 LncRNA and circRNA in CHD

LncRNAs are present throughout embryonic heart development [169], and research is increasingly focusing on their roles in CHD. Notably, the second most common cause of CHDs is deletion of 22q11.2, a region where the canonical Wnt/β-catenin signaling pathway plays a critical role in regulating heart outflow tract development. Indeed, aberrant activation of this pathway can result in abnormal embryonic heart development. Fa et al. found that lnc-TSSK2-8, located within the 22q11.2 region, activates canonical Wnt/β-catenin signaling and plays a crucial role in heart development through the action of small heat shock proteins HSPA6 and CRYAB [118,170]. Suppression of lncRNA NONMMUT063967.2 mitigates the impact of ASXL3 gene mutations, implicated in CHD [171], on cellular proliferation and apoptosis in murine cardiomyocytes [150]. Notably, MATR3, a nuclear matrix DNA/RNA-binding protein that is extremely prevalent in the fetal heart, interacts with pCharme, a chromatin-associated lncRNA expressed in developing mouse embryos and cardiomyocytes. Crucially, congenital abnormalities have been connected to mutations in MATR3. Maintaining the expression of developmental genes in fetal cardiomyocytes depends on the interaction between pCharme and MATR3 [151]. Ritter et al. meticulously delineated the crucial role of the indispensable lncRNA locus, Handsdown, in orchestrating developmental processes by precisely modulating cardiac gene expression programs during the formative stages of vertebrate embryogenesis [126]. Elevated Hand2 levels are the result of genetic ablation of the Handsdown locus, which mechanistically interacts with the Hand2 physically during cardiac differentiation. A gene called Hand2 is necessary for the development of the embryonic heart [172]. Han et al. showed that the full-length deletion of lncRNA Hand2os1/Uph, but not deletion promoter, in mouse triggers widespread upregulation of Hand2, causing marked biological effects, such as disrupted cardiac gene regulation, congenital heart defects, and perinatal death [173]. Furthermore, Upperhand, an lncRNA associated with hand2, sustains the super-enhancer signature and RNAPII elongation at the Hand2 enhancer, whereas its transcription blockade abolishes Hand2 expression, leading to right ventricular hypoplasia and embryonic lethality in mice [154].

There are also a few reports on the role of other ncRNAs in CHD. For instance, circRNA-105039, a downregulation circRNA in CHD, has been found to exert protective effects in induced pluripotent stem cells exposed to CHD by sponging miRNA-17, which targets cyclinD2 [174]. Liu et al. found that the content of circ-RCCD increases during heart development, as revealed by microarray analysis at embryonic days 13 and embryonic days 17. Furthermore, they demonstrated that by recruiting YY1 to the promoter region of MyD88, circ-RCCD exerts an inhibitory effect on MyD88 levels, which in turn promotes the differentiation of cardiomyocytes [156]. Emerging studies demonstrate that mitochondria-encoded circRNAs critically regulate mitochondrial functions. Recent work reveals their interaction with TNF receptor-associated protein 1, which promotes closure of the mitochondrial permeability transition pore to suppress deleterious ROS release. This mechanism underlies the therapeutic potential of exogenous mecciRNA in heart failure mouse models [117]. Recently, Wu et al. uncovered that transgenic cA-circSlc8a1, a circular antisense RNA, mice exhibited compromised heart function and, in severe cases, even developed hepatic steatosis. Mechanistically, cA-circSlc8a1 specifically silences circSlc8a1, a protective circular RNA that is highly expressed in the mouse heart during development, while not affecting its parental Slc8a1 linear mRNA. Specifically, circSlc8a1 exerts its cardioprotective effects by enhancing the translocation of mitochondria-associated proteins into the mitochondria and facilitating ATP production, which are crucial processes for maintaining normal heart function [175] (Fig. 4).

## 6 miRNA, lncRNA and circRNA as biomarkers in CHD

In the era of precision medicine, identifying efficient and specific biomarkers to guide early disease diagnosis, prognosis evaluation, and treatment monitoring has become one of the hotspots in medical research. Specific miRNAs have been found as potential biomarkers of CHD, such as ASD [176], VSD [177], AVSD [178], and TOF [179], in a host of clinical patient samples. For instance, plasma samples from individuals with CHD show a considerable upregulation of miR-8078, which is positively connected with pulmonary arterial hypertension. Plasma miR-8078 was identified by regression studies as an independent risk factor for CHD-PAH [180]. Compared to control patients, there was a significant difference in the expression levels of miR-221-3p, miR-21-5-5p, miR-26-5p, and miR-15-5p5 in CHD. Plasma levels of let-7a and let-7b are markedly elevated in ASD patients. When diagnosing ASD, the area under the ROC curve (AUC) value is between 0.833 and 0.900 [176]. Generally speaking, a higher AUC indicates greater diagnostic capability. Furthermore, cyanotic CHD patients had higher levels of miR-21-5p than non-cyanotic individuals [181], which is beneficial for identification of different CHD. Previously, a report exploring the underlying causes of VSD through the analysis of placental tissue emphasized the potential of microRNAs as biomarkers for diagnosing VSD [182]. This study not only elucidates a potential link between placental tissue and VSD but also hints at the pivotal role that the placenta may play in the pathogenesis of CHD broadly.

As high-throughput sequencing technology has advanced quickly in recent years, several lncRNAs have also been identified as possible CHD biomarkers. Yu et al. found that HOTAIR emerges as a potentially novel and valuable biomarker in the context of CHDs among patient populations [183]. In comparison to control groups, the expression of GATA3-AS1 and PWRN1 is dramatically downregulated in amniotic fluid from fetuses with ventricular septal defects, whereas the expression of LINC00598 and LINC01551 is significantly elevated [184]. Analogously, some circRNAs have also been identified as potential biomarkers for CHDs. For example, circ 0003416 expression is markedly downregulated in pediatric pulmonary arterial hypertension linked to CHD as compared to both the CHD group and the healthy control group [185].

The search for new circRNA biomarkers has seen a notable increase in enthusiasm and research efforts. In VSD cardiac tissue, circRNA microarray and qRT-PCR validation confirmed the upregulation of circRNA 002086 and downregulation of circRNA 007878, circRNA 101965, circRNA 402565, and circRNA 100709 [186]. CircRNA 0007798 appears to be significantly upregulated in myocardial tissues taken from people with TOF, according to new research, suggesting that it could be used as a potential diagnostic biomarker for this congenital heart abnormality [187]. Wu et al. utilized microarray expression analysis to identify three significantly down-regulated circRNAs, namely circRNA 079265, circRNA 004183, and circRNA 105039, in plasma samples obtained from individuals with CHD. These circRNAs had AUC values of 0.809, 0.758, and 0.907, respectively, indicating their potential diagnostic value. When combined, these three circRNAs yielded an AUC of 0.965, further emphasizing their strong association with CHD [188].

The identification, early diagnosis, prevention, and treatment process monitoring of CHDs rely on the discovery of a specific biomarker. Notably, while these studies have suggested that ncRNAs hold potential as biomarkers for CHD, they have not yet identified a specific biomarker. Therefore, more validation experiments, including animal models, mechanistic studies, and clinical trials, are necessary to better characterize the role of microRNAs in CHD. In addition, this section summarizes recent literature on miRNAs as biomarkers in CHD, aiming to provide a reference for researchers and clinicians in related fields. The most recent research on miRNAs, lncRNAs, and circRNAs as possible biomarkers for CHD is collected here (Table 2).

**Table 2:** ncRNA as biomarker in CHDs.

CHDs	miRNA	Sample	Regulation	AUC	Fold change	References
ASD	miR-20b-5p	Plasma samples	Upregulation	–	1.544	[158]
	has-let-7a	Plasma samples	Upregulation	0.833/0.917	–	[176]
	has-let-7b		Upregulation	0.900/0.680	–	
	hsa-miR-486		Upregulation	0.806/0.562	–	
	hsa-miR-221-3p	Cardiac biopsies	Downregulation	–	<0.1255	[189]
VSD	hsa-miR-218-5p		Upregulation	–	>19.350	[189]
	miR-1-3p	Amniotic fluid	Upregulation	–	4.010	[190]
	miR-206-3p		Upregulation	–	3.876	
	miR-1b		Upregulation	–	2.343	
	miR-184		Upregulation	–	3.048	
	miR-15b-5p		Upregulation	–	2.365	
	miR-293-5p	Maternal serum	Upregulation	–	3.069	
	miR-208b-3p		Downregulation	–	0.188	
	miR-877		Upregulation	–	2.272	
	miR-433-3p		Upregulation	–	2.255	
	miR-206-3p		Upregulation	–	2.043	
	miR-1-3p	Myocardial tissue	Downregulation	–	0.437	
	miR-1b		Downregulation	–	0.491	
	miR-293-5p		Downregulation	–	0.457	
	miR-185-5p		Downregulation	–	0.349	
	miR-96-5p		Upregulation	–	4.068	
	miR-497-5p	Amniotic fluid-derived exosomal samples	Upregulation	–	–	[191]
	miR-144-3p		Downregulation	–	–	
	miR-129-5p		Downregulation	–	–	
	LINC00598	Amniotic fluid	Downregulation	–	2.296	[184]
	GATA3-AS1		Upregulation	–	2.222	
	PWRN1		Upregulation	–	2.096	
	LINC01551		Downregulation	–	2.059	
	hsa_circRNA_002086	Heart tissue	Upregulation	–	55.976	[186]
	hsa_circRNA_007878		Downregulation	–	56.333	
	hsa_circRNA_100709		Downregulation	–	312.080	
	hsa_circRNA_101965		Downregulation	–	37.822	
	hsa_circRNA_402565		Downregulation	–	53.329	
TOF	miR-222	Right ventricle tissues	Upregulation	–	–	[160]
	LncRNA TBX5‐AS1:2	Foetal hearts tissue	Downregulation	–	5.612	[161]
	hsa_circ_0007798	Foetal heart tissue	Upregulation	–	2.668	[187]
CHD	miR-375-3p	Maternal serum	Upregulation		–	[192]
	N4bp2l2	Heart tissue (rats)	Upregulation		58.65	[193]
	Atp9b		Upregulation		44.23	
	Atp2b1		Downregulation		0.011	
	Hace1		Downregulation		0.022	
	HOTAIR	Plasma and tissue	Upregulation	–	–	[183]
	ENST00000436681	plasma	Upregulation	0.892	179.501	[194]
	ENST00000422826		Upregulation	0.817	41.989	
	AA584040		Downregulation	0.755	392.765	
	AA709223		Downregulation	0.882	229.905	
	BX478947		Downregulation	0.886	26.765	
	hsa_circRNA_004183	Plasma	Downregulation	0.758	–	[188]
	hsa_circRNA_079265		Downregulation	0.809	–	
	hsa_circRNA_105039		Downregulation	0.907	–	

## 7 The function of ncRNA in placenta

While previous reports have indicated the potential role of ncRNAs in CHD, they have consistently fallen short in providing a systematic synthesis of this field. Crucially, these studies have largely overlooked the critical contributions of ncRNAs during placental development, a process whose profound importance for CHD pathogenesis remains underexplored [164,169,195]. We have comprehensively summarized the roles and potential mechanisms of miRNA, lncRNAs and circRNAs in regulating placental development or participating in the related diseases mediated by abnormal placental development in recent years (Table 3).

**Table 3:** The function of ncRNA in placenta development.

ncRNA name	Animal model	Target(s)	Function	Related diseases	References
miR-1290	In vitro	LHX6	Placental trophoblast cell-derived exosomal miR-1290 promote the transformation of endometrial cells to promote implantation.	–	[196]
miR-378a-3p	In vitro	Caspase-3	Inhibit decidual cell apoptosis and promote proliferation	EPL	[197]
miR-455-3p	In vitro	NFAT5	Downregulation of miR-455-3p in decidual cells promotes macrophage polarization and suppresses trophoblasts invasion	PE	[198]
miR-30d-5p	In vitro	HDAC9	Placenta-derived exosomes miR-30d-5p polarize macrophages and modulate trophoblast and endothelial cell functions.	–	[199]
miR-21-5p	Both	SMAD7	Exogenous miR-21-5p regulate trophoblast cell viability, proliferation and motility	SA	[200]
miR-3928-3p	In vitro	CCL3	Hsa-miR-3928–3p knockdown increases CCL3 expression induce hAECs senescence via the CCR5 receptor.	–	[201]
miR-205	Both	IL-32	Regulate trophoblast proliferation, migration, invasion and tube formation ability of HUVECs	Pregnancy-induced hypertension	[202]
miR-377-3p	Both	NR6A1	Regulate placental immune function	CHD	[203]
miR-424-5p	In vitro	LRP6	suppressed trophoblast proliferation, migration, and invasion, as well as the tube formation of HUVECs	Placenta accreta	[204]
miR-214-3p	Both	PlGF/eNOS	Influence trophoblast invasion and endothelial dysfunction	PE	[205]
miR-510-3p	Both	VEGFA	Impaired trophoblast function and vascular dysfunction	PE	[206]
miR-22-5p_R-1	Both	PDK4	Inhibit trophoblast metabolic switch and EMT	RSA	[207]
miR-410-5p	Both	STAT1	Regulate placental immune function	SA	[208]
miR-410-3p	Both	TRAF6	Inhibit trophoblast EMT, migration and invasion	SA	[209]
miR-146a-5p	Both	TRAF6	rescue inflammation and inhibit apoptosis	Antiphospholipid syndrome	[210]
miR-20b-5p	In vitro	TG16L1 and ATG7	Inhibit trophoblast cell invasion and autophagy	RA	[211]
miR-135b-5p	In vitro	ADAM12	Promoted apoptosis and inhibited trophoblast cell invasion and migration	PE	[212]
miR-146b-5p	Both	IRAK1 and ADAM19	Suppress trophoblast proliferation, invasion, migration, and implantation-associated inflammation	Miscarriage	[213]
miR-135a-5p	In vitro	SIRT1	Encouraged trophoblast cell growth, invasion and migration	GDM	[214]
miR-526b-5p	In vitro	c-Myc and Foxp1	Regulate the proliferation, migration, and invasion of trophoblasts	RSA	[215]
miR-95-3p	In vitro	EPM2A	Promote the migration and invasion of trophoblast	PE	[216]
MALAT1	In vitro	miR-424	Regulate the biological functions of trophoblasts and endothelial cell angiogenesis	sFGR	[217]
Lnc MALAT1	Both	miR-133a-3p	Modulates placental trophoblast function	PE	[218]
Lnc HZ08	Both	FOXA1/CtIP/BRCA1;	Suppress homologous recombinati repair in trophoblast cells	Unexplained miscarriage	[219]
Lnc HZ06	Both	IL1B	Suppresses migration, invasion and promotes apoptosis of human trophoblast cells	RA	[220]
Lnc HZ14	Both	ZBP1/NLRP3	Promotes trophoblast cell pyroptosis	Miscarriage	[221]
Lnc HZ09	Both	PLD1	Regulate trophoblast cell migration and invasion and affect the occurrence of miscarriage	RA	[222]
Lnc DUXAP8	Both	PCBP2	Regulate endoplasmic reticulum selective autophagy	PE	[223]
Lnc DUXAP8	In vitro	EZH2/SUZ12/LSD1	Promoted trophoblast cell proliferation, migration and invasion and regulate cell cycle and apoptosis of trophoblasts	PE	[224]
Lnc SNHG12	Both	Dio2	Regulate the invasion and migration of trophoblasts	RSA	[225]
Lnc HZ12	Both	BBC3	Regulate placenta apoptosis	Miscarriage	[226]
Lnc NEAT1	In vitro	miR-217	Inhibit trophoblast cell migration and invasion	PE	[227]
Lnc UCA1	In vitro	IFITM3	Influence hTSC proliferation and trophoblast syncytialization	Placental syndromes	[228]
Lnc HZ06	Both	HIF1α-SUMO	Causes trophoblast cell ferroptosis	Miscarriage	[229]
Lnc XIST	Both	miR-497-5p	Regulate trophoblast proliferation, migration, apoptosis and cell cycle arrest	GDM	[230]
Lnc HOXD-AS1	In vitro	miR-135a	Regulate trophoblast phenotype and the secretion of inflammatory factors	PE	[231]
Lnc AOC4P	In vitro	TRAF6	Inhibit the glycolysis of trophoblast cells and M2 polarization of macrophages	RSA	[232]
Lnc GHET1	In vitro	EZH2/LSD1	regulates extravillous trophoblastic phenotype	PE	[233]
Lnc TDRKH-AS1	Both	PDIA4	initiates endothelial cells pyroptosis	PE	[234]
Lnc TLR8-AS1	Both	TLR8/STAT1	Inhibit the proliferation, migration and invasion of trophoblast cells	PE	[235]
Lnc BBOX1-AS1	In vitro	hnRNPK	Inhibite proliferation, migration, invasion, tube formation and promoted apoptosis of trophoblast cells	Recurrent pregnancy loss	[236]
Circ ADAM9	Both	miR-375	Circ-ADAM9 downregulation mitigates placental injury	GDM	[237]
Circ PCNXL2	In vitro	miR-487a-3p	Inhibit proliferation and migration of trophoblast cells	PE	[238]
Circ 0081343	In vitro	Rbm8a	Activate trophoblast autophagy	FGR	[239]
Circ CULT1	Both	miR-30e-3p/ANXA1	Promote autophagy while concurrently suppressing trophoblast cell proliferation, migration, and invasion.	FGR	[240]
Circ 0007611	In vitro	miR-34c-5p	Enhance trophoblast apoptosis and inhibit proliferation	PE	[241]
Circ PAPPA2	In vitro	miR-942/miR-5006-3p	Regulate trophoblast proliferation and invasion	PE	[242]
Circ 0007445	In vitro	miR-4432	Regulate trophoblast proliferation, migration and invasion	PE	[243]
Circ AMN1	Both	miR-205_R-1	Promote trophoblast ferroptosis	Retained placenta	[244]
Circ THBS1	In vitro	miR-136-3p	Modify the biological function of trophoblast cells	FGR	[245]
Circ 0001861	In vitro	miR-296-5p	Modify the biological function of trophoblast cells	PE	[246]
Circ 0002348	Both	miR-126-3p	Inhibit trophoblast proliferation and promote trophoblast apoptosis	PE	[247]
Circ CRIM1	In vitro	miR-942-5p	Regulate trophoblast proliferation, migration, and invasion	PE	[248]
Circ PTK2	In vitro	miR-619	Regulate trophoblast proliferation and migration	PE	[249]
Circ MPP1	Both	YTHDC1	Regulate the function of placental trophoblast cells	placental dysfunction	[250]
Circ 0001326	In vitro	miR-145-5p	Regulate trophoblast cell phenotype alteration	PE	[251]
Circ 0001326	In vitro	miR-188-3p	Regulate trophoblast cell phenotype alteration	PE	[252]
Circ 0004904	In vitro	miR-19a-3p	Reduced trophoblast cells proliferation and motility	PE	[253]
Circ 0111277	In vitro	miR-188-3p	Inhibit trophoblast cell phenotype function	PE	[254]

### 7.1 MicroRNA in placenta

Following fertilization, the blastocyst emerges as the first visibly distinct stage, characterized by two morphologically distinct cell populations [255]. Specifically, it comprises a trophectoderm layer encircling its periphery, destined to evolve into the placenta, and an inner cellular mass residing within its cavity, which serves as the precursor for both the embryo and the visceral endoderm [256]. During the ontogenesis of the human placenta, trophoblast cells undergo differentiation along two distinct yet interconnected pathways. In the villous trajectory, cytotrophoblast cells undergo a process of fusion, ultimately giving rise to the formation of multinucleated syncytiotrophoblast, which plays a pivotal role in nutrient exchange and fetal development. Alternatively, cytotrophoblast cells evolve into either endovascular or interstitial extravillous trophoblasts along the extravillous pathway, exhibiting an invasive behavior. These specialized cells penetrate into the maternal decidua and uterine vessels, respectively, facilitating placental implantation and remodeling of the maternal vasculature, thereby ensuring a successful pregnancy [257,258].

It is important to note that the proper characteristics and functions of various trophoblast subtypes in the placenta throughout the early phases of development may be crucial in determining how the fetus's organs develop later on [259]. Recent studies have shown the complex regulatory function of ncRNA in the placenta on the proliferation, migration, and immunological responses of specific trophoblasts, based on this crucial knowledge [260]. For instance, the miRNA-290 and miRNA-322 clusters modify the expression of trophoblast stem cell hallmark transcription factors, which in turn impacts cell proliferation and differentiation [261]. The TNF receptor-associated factor 6 expression was directly repressed at the post-transcriptional level by miR-146a-5p and miR-146b-5p, which prevented trophoblast EMT and reduced their capacity for migration and invasion in vitro [262]. Recent research has uncovered two distinct effects of miRNA 218-5p in vascular remodeling in part via inhibition of TGF-β2 signaling. Firstly, it accelerates the remodeling of placental spiral artery and recruitment of leukocytes at the remodeling site. Secondly, meanwhile it leads to a substantial increase in the depth of trophoblast invasion and promotes trophoblast vascular transformation, both of which are observed to double in comparison to control conditions [263].

Apart from trophoblast cells, miRNAs also demonstrate their regulatory roles in other types cells, such as decidual cells, maternal vascular endothelial cells, embryonic stem cells and human umbilical vein endothelial cells, associated with the placenta development. For instance, overexpression of miRNA-378a-3p, which directly binds to the 3′-UTRs of Caspase-3, inhibits its expression, thereby suppressing the apoptosis of decidual cells [197]. Decidual cells are crucial components in the development of the placenta and fetus, supporting placental attachment while simultaneously participating in immune regulation. Ma, C. et al. discovered that decidual cells' downregulation of miR-455-3p reduces trophoblast invasion and encourages macrophage polarization [198]. Targeting PDK4, exosomes produced from decidual stromal cells release miRNA-22-5p-R-1 to inhibit the trophoblast epithelial-mesenchymal transition and metabolic shift from mitochondrial respiration to glycolysis [264]. Human umbilical vein endothelial cells play a pivotal role in the formation and remodeling of placental vasculature, with their health status directly influencing the circulatory and material exchange functions of the placenta. Liu, R. et al. showed that miRNA-320a targets the estrogen-related receptor gamma to control the biological activity of trophoblasts and endothelial cells [265]. Recently, Zhang, L. et al. have shown that miRNA-140-3p and miRNA-574-3p exert inhibitory effects on umbilical vein endothelial cells (UVECs) by specifically targeting vascular endothelial growth factor (VEGF), thereby suppressing their proliferation, migration, and capacity for tube formation [266]. By fine-tuning these cellular processes, ncRNAs exert a profound influence on the placental microenvironment/development, ultimately affecting the orchestrated development and function of fetus organ [267,268].

In addition to their direct effects on placental trophoblast biology, miRNA may also influence fetal organogenesis indirectly by modulating the placental secretion of hormones, growth factors, and cytokines. These factors are critical for fetal growth and organ development, and their dysregulation can lead to congenital abnormalities and other complications. For instance, miRNA181a-5p and miRNA-663 play a pivotal role in regulating the human placental (pro)renin receptor-prorenin-angiotensin system, which is a critical regulator of placental function, such as controlling trophoblast proliferation, angiogenesis and blood flow [[269], [270], [271]]. By controlling the expression of angiogenic factors like VEGF, miRNAs may have an effect on the development of the placenta's vascular network. For example, miRNA-203 inversely correlates with, while VEGFA and VEGFR2 positively correlate with, gestational age in placenta. Furthermore, miRNA-203 has been found to inhibit the expression of both VEGFA and VEGFR2 in HUVECs, suggesting a potential regulatory role in placental development [272]. However, there is no in vivo research to definitively show that miRNA-203 regulates placental angiogenesis, nor is there any direct evidence, such as luciferase reporter test, to indicate the relationship between miRNA-203 and VEGFA/VEGFR2. Notably, Zhu, Y. et al. discovered that abnormal miRNA-16 levels in pregnant mice substantially affect the placenta and embryo weights, change the number of offspring, and modify microvascular density. Mechanistically, miRNA-16 controlled placental angiogenesis and development by directly inhibiting VEGF expression by precisely targeting a certain sequence inside its 3′-untranslated region of its mRNA [273]. The regulation of placenta development by miRNA through cytokines affecting the placenta is also a common phenomenon. Recent research indicates that by post-transcriptionally targeting the cytokine TNF receptor-associated factor 6, miRNA-410-3P from the serum of patients who suffered spontaneous miscarriage suppressed trophoblast cell invasion, migration, and EMT [274]. Additionally, new data indicates that the PI3K/Akt and MAPK pathways, which are known to influence cellular responses to growth stimuli, are probably involved in the control of fetal growth and development by miRNA [274,275] (Fig. 4).

### 7.2 LncRNAs and circRNA in placenta

LncRNAs and circRNAs play complex and subtle regulatory functions in placenta formation and production. Recent study has found that MALAT1 downregulation, via miR-424 competitive binding, reduces ERRγ and HSD17B1 expression, impairing trophoblast functions including proangiogenic activity, invasion, migration, and hypoxic adaptation [217]. By reducing BRCA1-mediated CtIP ubiquitination, upregulating FOXA1, and interfering with BRCA1-CtIP interactions, Lnc-HZ08 prevents homologous recombination repair in trophoblast cells [219]. Deficiencies in homologous recombination repair mechanism result in the inability of mammalian cells to accurately and effectively repair double-strand breaks which could induce genomic instability, chromosomal aberration, cell death, and even CHD [276,277]. By downregulating HSPA8 and LAMP2A, Lnc-HZ12, which was found to be significantly overexpressed in the villous tissues of patients who experienced repeated miscarriages, inhibits the CMA degradation of BBC3 in trophoblast cells. It also competitively binds with BBC3 to hinder the formation of complexes, which ultimately promotes trophoblast cell apoptosis and is linked to miscarriage [278].

CircRNA 0081343 sponges Rbm8a in cytoplasm, promoting its nuclear translocation via Ipo13 and activating trophoblast autophagy, thereby regulating trophoblast cells [279]. circCUL1, interacting with miRNA-30e-3p, regulates trophoblast autophagy via ANXA1/PI3K/AKT, which significantly impacts placental development speed and weight in mice [275]. A type of programmed cell death called ferroptosis can control the growth of an organism by influencing the invasion and proliferation of trophoblast cells [280]. Lv et al. discovered that circAMN1 controls ferroptosis in trophoblast cells by acting as a miR-205 R-1 sponge [244]. By preventing trophoblast cell invasion and proliferation through the miR-487a-3p/interferon regulatory factor 2 axis, CircPCNXL2 may accelerate the development of preeclampsia [281], which has been linked to an increased risk of developing CHD. The interaction between CircHIPK3 and miR-124 influences placental dysfunction and disrupts endothelial cell angiogenesis in early-onset preeclampsia, a process that is mediated by CPT1A-facilitated fatty acid oxidation [282] (Fig. 4).

## 8 Conclusion and prospects

**Fig. 5:**
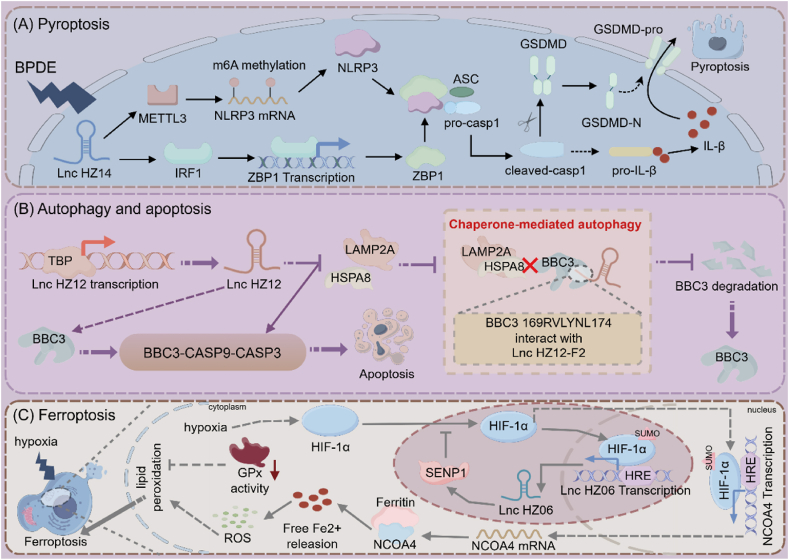
**Mechanisms of different cell death modes mediated by ncRNAs in placenta development.** (A) The schematic diagram of lnc HZ14-mediated trophoblast cell pyroptosis [221]. (B) The schematic diagram of lnc HZ12-mediated trophoblast cell autophagy and apoptosis [278]. (C) The mechanism diagram of lnc HZ06-mediated trophoblast cell ferroptosis [229].

In summary, ncRNAs significantly modulate the development of both CHD and placenta In the context of CHD, ncRNAs exert significant influence on cardiac development and disease progression by participating in gene expression regulation, post-transcriptional modification, and cellular signal conduction. Notably, distinct modes of cell death regulation, which have been extensively reviewed in relation to ncRNAs in cardiovascular diseases, also play crucial roles in this process (Fig. 5). Additionally, several ncRNA molecules have been found to be important regulators linked to CHD, offering new targets for the diagnosis and management of the condition. Concurrently, ncRNAs play indispensable roles in placental development. The majority of current research focuses on how they control trophoblast cell proliferation, differentiation, and invasion, which preserve the placenta's normal shape and function. Additionally, the regulation of ncRNAs on the hormones, growth factors, oxygen, blood flow, and nutrient supply secreted by the placenta, or the transmission of genetic material, will further affect the development of embryonic organs. Unfortunately, there is a lack of research in these aspects. Moreover, the abnormal expression or dysfunction of ncRNAs may lead to abnormal placenta development, thereby triggering a series of pregnancy complications. Therefore, ncRNAs as biomarkers for abnormal placenta function are also a future research direction.

Future research endeavors in elucidating the specific mechanisms of ncRNAs, especially piRNA, transfer RNA-derived small RNAs, snoRNA and so on, in heart and placental development, especially in the direction of the fetal heart axis, will undoubtedly open up a series of new scientific explorations. NcRNAs act not merely as precise regulators of gene expression but also as integral components of complex regulatory systems involving DNA, mRNA, proteins, and other biomolecules that collectively orchestrate developmental processes. Thus, a deeper understanding of the interaction between ncRNAs and other biomolecules, or the epigenetic regulatory mechanisms they mediate, will yield more comprehensive understanding of these vital processes.

In terms of mechanism research, future work may include, but is not limited to: utilizing high-throughput sequencing technology combined with bioinformatics analysis to identify more ncRNAs related to CHD and placental development; constructing animal models with ncRNA knockout or overexpression using CRISPR/Cas9 and other gene-editing technologies to observe their impact on heart and placental development; employing RNA-Seq, ChIP-Seq, and other techniques to decipher the interaction networks between ncRNAs and DNA, proteins, and other molecules, thereby revealing the specific mechanisms underlying their regulation of heart and placental development.

Moreover, the development of specific therapeutic strategies targeting ncRNAs will be another major focus of future research. With the increasing maturity of gene therapy technologies, the precise regulation of specific ncRNAs using antisense oligonucleotides, CRISPR/Cas9, and other techniques holds promise as a novel approach for treating CHD and placental developmental abnormalities. Simultaneously, the development of small molecule inhibitors will also provide new options for ncRNA-targeted therapy. These inhibitors may interfere with the interaction between ncRNAs and other molecules by binding to ncRNAs, thereby regulating ncRNA function.

## CRediT authorship contribution statement

**Haoxuan Wang:** Writing – original draft, Software, Methodology, Investigation, Formal analysis, Data curation. **Xinzhe Chen:** Methodology, Investigation, Formal analysis. **Yinghui Li:** Methodology, Investigation, Formal analysis. **Shudan Xiao:** Investigation, Formal analysis. **Tianqi Teng:** Investigation, Formal analysis. **Sumin Yang:** Methodology, Investigation. **Kun Wang:** Writing – review & editing, Funding acquisition, Conceptualization. **Meihua Zhang:** Conceptualization, Methodology, Investigation.

## Availability of data and materials

The data that support the findings of this study are available from the corresponding author upon reasonable request.

## Funding

This work was supported by Qingdao Science and Technology Benefiting the People Demonstration Project (24-1-8-smjk-7-nsh); Open Project Program of State Key Laboratory of Frigid Zone Cardiovascular Diseases (SKLFZCD), Harbin Medical University (HDHY2024007); National Natural Science Foundation of China (82370291, 82070313) and Major Basic Research Projects in Shandong Province (ZR2024ZD46).

## Declaration of competing interest

The authors declare that they have no known competing financial interests or personal relationships that could have appeared to influence the work reported in this paper.

## **Abbreviation**

ncRNAs: Non-coding RNAs
CHD: Congenital heart disease
TOF: Tetralogy of fallot
VSD: Ventricular septal defect
ASD: Atrial septal defect
miRNA: MicroRNA
lncRNA: Long ncRNA
circRNA: Circular RNA
nt: nucleotides
Pol: Polymerase
miRISC: MiRNA-induced silencing complex
kb: Kilobases
3′ UTR: 3′ untranslated region
PRC2: Polycomb repression complex 2
ceRNA: Competing endogenous RNA
ciRS-7: Circular RNA sponge for miR-7
ciRNAs: Circular intronic RNAs
EIciRNAs: Exon-intron circular RNAs
snRNP: Small nuclear ribonucleoprotein
snRNA: Small nuclear RNA
Hand1: Heart and neural crest derivatives-expressed 1
Mesp1: Mesoderm posterior 1
EVT: Extravillous trophoblasts
UVECs: Umbilical vein endothelial cells
VEGF: Vascular endothelial growth factor
EPL,: Early pregnancy loss
PE: Preeclampsia
SA: Spontaneous abortion
PA: Placenta accreta
RA: Recurrent abortion
GDM: Gestational diabetes mellitus
RSA: Recurrent spontaneous abortion
